# Ginsenoside Rg3 inhibits angiogenesis in a rat model of endometriosis through the VEGFR-2-mediated PI3K/Akt/mTOR signaling pathway

**DOI:** 10.1371/journal.pone.0186520

**Published:** 2017-11-15

**Authors:** Yang Cao, Qing Ye, Mengfei Zhuang, Shuwu Xie, Ruihua Zhong, Jingang Cui, Jieyun Zhou, Yan Zhu, Tingting Zhang, Lin Cao

**Affiliations:** 1 Department of Gynecology, Yueyang Hospital of Integrated Traditional Chinese and Western Medicine, Shanghai University of Traditional Chinese Medicine, Shanghai, China; 2 Department of Neurology, Longhua Hospital, Shanghai University of Traditional Chinese Medicine, Shanghai, China; 3 Department of Reproductive Pharmacology, NPFPC Key Laboratory of Contraceptives and Devices, Shanghai Institute of Planned Parenthood Research, Shanghai, China; 4 Clinical Research Institute of Integrative Medicine, Shanghai University of Traditional Chinese Medicine, Shanghai, China; Rutgers University, UNITED STATES

## Abstract

**Objective:**

This study aimed to investigate the link between the inhibitory effect of ginsenoside Rg3 on the ectopic endometrium growth and the VEGFR-2-mediated PI3K/Akt/mTOR signaling pathway, a mechanism known to inhibit angiogenesis and induce ectopic endometrial cell apoptosis.

**Materials and methods:**

A model of endometriosis was established by allotransplantation in rats. The rats were randomly divided into 5 groups: the ginsenoside Rg3 low-dose group (group A,5mg/kgBW/d of ginsenoside Rg3), the ginsenoside Rg3 high-dose group (group B, 10mg/kgBW/d of ginsenoside Rg3), the gestrinone group (group C, 0.5mg/kgBW/d of gestrinone), the control group (groupD, 10ml/kg BW/d of 0.5%CMC-Na) and the ovariectomized group (group E, 10ml/kgBW/d of 0.5%CMC-Na). Rats were executed after 21 days of continuous administration. The ectopic endometrium volume was measured and the inhibitory rate was calculated. The levels of serum estradiol (E_2_) and progesterone (P) were detected by Electro-Chemiluminescence Immunoassay (ECLI). The protein expressionof VEGF, VEGFR-2, p-Akt, and p-mTOR inthe ectopic endometrium wastested by immunohistochemistry(IHC) and Western Blotting. The mRNA expression levels of VEGF, VEGFR-2, Akt, and mTOR were tested by Real-Time Polymerase Chain Reaction (PCR). The apoptosis rate of the ectopic endometrial cells was detected by Terminal Deoxynucleotidyl Transferase-mediated Digoxigenin-dUTP Nick-End Labeling Assay(TUNEL).

**Main results:**

Tissue measurements revealed a dose-dependent inhibition effect of ginsenoside Rg3 on the growth of the ectopic endometrium in treated rats compared to controls. Immunohistochemical and Western Blotting assays confirmed that the expression of VEGF, p-Akt, and p-mTOR was down-regulated in ginsenoside Rg3 -treated lesions. Real-time PCR results also showed that the mRNA expression levels of VEGF, Akt, and mTOR in the ectopic endometrium were reduced.

**Conclusions:**

The present study demonstrates, for the first time, that ginsenoside Rg3 suppresses angiogenesis in developing endometrial lesions. The ginsenoside Rg3 inhibitory effect on the growth of the ectopic endometrium in EMs rats might occur through the blocking of the VEGFR-2-mediated PI3K/Akt/mTOR signaling pathway, thus halting angiogenesis and promoting the apoptosis of ectopic endometrial cells.

## Introduction

Endometriosis(EMs)is a frequent disease, which affects at least 10% of women during their reproductive life. The incidence of EMs among infertile women is approximately 40%. Of the affected women, approximately 90% experience pelvic pain[1]. EMs is known to be the primary cause of dysmenorrhea, pelvic pain, and infertility. Although EMs is a benign lesion, its invasive nature, the rate of metastasis and recurrence are typical of a clinically malignant lesion and thus, it has been generally referred to as benign cancer. The 5-year recurrence rate of EMs is above 40%, whether treated with surgery or drug therapy [2], and the cancer occurs clinically in 0.7 to 1.6% of patients in a 8-year follow-up [3]. The exact pathogenesis of EMs is not clear, with most scholars recognizing Sampson's Theory of Implantation of Endometriosis, which postulates that EMs is caused by backflow menstruation. It is generally accepted that the formation and growth of ectopic lesions require the supply of oxygen and nutrients. Studies have indicated that angiogenesis is an important feature of EMs, however, the underlying mechanism of angiogenesis in EMs remains unclear [4].

The treatment of EMs is mainly based on surgery and drug therapy. Surgery can remove ectopic foci, recover normal pelvic anatomy, relieve pain, and increase the chances of pregnancy. Drug therapy aims to inhibit the ovarian hormone secretion, but the effect is short-lived and has side effects such as the onset of menopausal symptoms and osteoporosis [5]. Regardless of treatment, EMs has a high recurrence rate. Therefore, new treatments for EMs aimed at reducing the recurrence rate and recovering fertility have become a current focus on EMs research. Recent studies investigated the molecular mechanism of EMs and have provided new treatment possibilities, especially focused on anti-angiogenesis strategies [6].

After menstrual blood anarrhea into the abdominal cavity, the growth of the ectopic endometrium requires adequate nutrient supply; therefore neovascularization is key to the survival of the ectopic endometrium [7]. Vascular endothelial growth factor (VEGF), the strongest angiogenic factor, can specifically combine with vascular endothelium to promote endothelial proliferation and angiogenesis and can increase vascular permeability. Some studies indicate that anti-VEGF/VEGFR agents appeared to inhibit the growth of endometriosis, with no effect on ovarian function, and therefore anti-angiogenic therapy may become a future strategy in treating endometriosis [8]. VEGF and its receptor-mediated signal transduction pathways play an important role in physiological and pathological angiogenesis. Among them, the phosphatidylinositol 3-kinase/protein kinase B/mammalian target of rapamycin (PI3K/Akt/mTOR) signaling pathway plays an important role in the occurrence and development of a variety of tumors. ThePI3K/Akt/mTOR pathway promotes tumor angiogenesis by altering the activation state of a large number of downstream effector molecules and inducing cell proliferation and inhibiting apoptosis to further promote tumor development. Activated mTOR and other kinases can induce4E-BP1 phosphorylation and reduce the affinity of 4E-BP1 and elF-4E. Released elF-4E combines with elF-4G, -4B, and -4A, and then translates encoded cyclinD1, HIF1 alpha and, subsequently, VEGF [9]. This seems to indicate that not only is VEGF an activator of the PI3K/Akt signaling pathway, but also the activation of the PI3K/Akt signaling pathway is itself promoted by the transcription of VEGF in a positive feedback loop. At present, the inhibition of expression of PI3K, Akt and other related genes by gene knockout or small molecule drugs towards the blocking of the activation of a variety of downstream anti-effector molecules, the inhibition of angiogenesis and induction of apoptosis, has become the focus of tumor therapy research.

Ginsenoside Rg3 is the main effective component of ginseng. It is the fourth ring of three saponins of the Panax ginseng saponin monomer, extracted from red ginseng by the famous Japanese natural medicine chemist, Kitagawa Hoon, in 1980. ginsenoside Rg3’s extraction rate is 0.003%, the molecular formula is C_42_H_72_O_13_, and the molecular weight is 785.01. As a vasodepressor, ginsenosideRg3 was the first compound to be used for anti-tumor treatment. Its way of action involved the prevention of tumor cell secretion of vascular growth factors, interfering with the interaction between endothelial cells and the extracellular matrix, and reducing the microvessel density in tumor tissue. The use of ginsenoside Rg3 had no significant effect on sex hormone levels and thus, no estrogen deficiency associated side effects. Ginsenoside Rg3 is the only tumor angiogenesis inhibitor with clinical application in the world. At present, ginsenoside Rg3 is mainly used to inhibit the recurrence and metastasis of various malignant tumors after surgery, radiotherapy, and chemotherapy. It can inhibit the proliferation of tumor vascular endothelial cells and the formation of new blood vessels by regulating some cytokine anti-angiogenic factors[10]. Recently, Chinese researchers have found that ginsenoside Rg3 can inhibit the development of endometriosis lesions in rats, reduce the volume of ectopic lesions, inhibit the expression of VEGF, and halter angiogenesis[11]. However, the effect of the ginsenoside Rg3 on VEGFR-2 and associated signal transduction pathway remained unclear. Therefore, this study aims to investigate the effect of ginsenoside Rg3 on EMs treatment in rats and to investigate the interaction between the ginsenoside Rg3 and the VEGFR-2 mediated PI3K/Akt/mTOR signaling pathway. Ultimately, this study will contribute to the development of a new method for early diagnosis and prognosis of clinical EMs based on the administration of an angiogenesis inhibitor.

## Materials and methods

### Animals

This study used female Sprague-Dawley (SD) rats, 6–7 weeks old, weighing between 180 and 200g each, raised in the Laboratory Animal Center of the Shanghai Institute of Planned Parenthood Research(Certificate of Quality No: SCXK [hu] 2013–0016). All animal tests were conducted in accordance with the AVMA Guidelines for the Euthanasia of Animals: 2013 Edition[12]. The study was approved by the Animal Care and Use Committee of the Shanghai Institute of Planned Parenthood Research (number 2015–04). The rat’s sample size was calculated according to Wei (2010)[13].

### Chemicals and reagents

**Fig 1:**
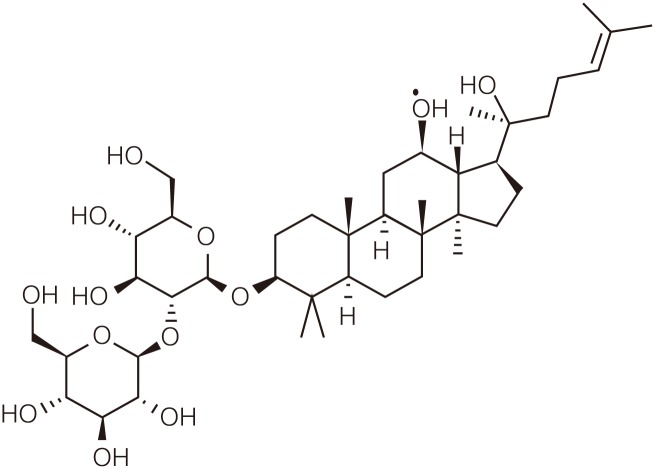
Molecular structure of the ginsenoside Rg3.

GinsenosideRg3((3-beta,12-beta)-12,20-Dihydroxydammar-24-en-3-yl2-O-beta-D -glucopyranosyl-beta-D-glucopyranoside, C_42_H_72_O_13_, molecular weight 785.01,purity>95%) were kindly donated by the Shanghai Shengzhong Pharm&ChemCo.,Ltd (Shanghai, China). Its molecular structure is shown in Fig 1. Gestrinone was purchased from Beijing ZiZhu Pharmaceutical (Beijing, China). Estrogen and Progesterone ECLI kits were purchased from Roche (Switzerland). The polyclonal rabbit anti-VEGF, anti-VEGF receptor 2 and the monoclonal rabbit anti-AKT1,anti-p-AKT1 (S473), and anti-mTOR were all purchased from Abcam (Cambridge, UK). The monoclonal rabbit anti-p-mTOR (Ser2448)was purchased from Cell Signaling Technology (Boston,MA,USA). The apoptosiskit was purchased from Roche(Switzerland).

### Rat model

All operational processes were conducted under sterile conditions according to the procedures described in Jones[14]. The rats were anesthetized with 3% pentobarbital sodium before a vertical incision in the abdomen was made for tumor transplantation. All parts of the uterus were removed and immediately placed in a saline solution. The endometria were separated from the myometrium and cut into 0.5×0.5 cm pieces. The uterine segments were sutured onto the peritoneum close to blood vessels. The incision was closed and disinfected, and the animals were allowed to recover from anesthesia. Estradiol benzoate was subcutaneously injected for 3 days following the operation.

### Experimental design and sampling of biological materials

Tissue from the endometrial lesions was obtained on day 21 after transplantation. The volume (length×width×height) was measured using an electronic digital caliper. Rats with a volume of ectopic tissue larger than 20 mm^3^ were randomly divided into 6 groups by weight (S1 Table).

After 21 days, blood was drawn from the abdominal aorta under anesthesia, and then the rats were executed with narcotic overdose and dissected. A 2.5 ml blood sample was taken and centrifuged, the serum was extracted and kept at −20°C. The ectopic endometrium was removed, and tissues were partly fixed in a 4% neutral paraformaldehyde solution and partly frozen in liquid nitrogen.

### Electro-Chemiluminescence Immunoassay (ECLI)

Blood was centrifuged at 3000r/minfor10min, and then the supernatant fluid was removed. The ECLI’s assay was performed and the concentrations of E_2_ and P in serum were determined according to manufactures instructions (Roche, Switzerland).

### ImmunohistochemicalStaining

Rats’ ectopic endometria fixed in a 4% neutral paraformaldehyde solution, were washed, dehydrated, waxed, and embedded. Slides, soaked in a wash buffer over-night, were rinsed with running water and dried. After being immersed in 100 pg/ml polylysine for 30 min at 37°C, the slides were inserted into a clean glass shelf overnight at 37°C. A microtome was used to cut 4-μm-thick serial sections, and 5 of these sections were selected to sit for 1 h at 56°C and then over-night at 37°C. Before staining, the sections were placed in a 60°Cwarm case for 1 h. Sections were then dewaxed in xylene and rehydrated, followed by microwaving in 10 mM sodium citrate buffer (pH 6.0) for antigen retrieval. The sections were washed three times with PBS (0.01 mmol/L, pH 7.4) for 3 min. The immunohistochemistry S-P method was conducted according to the manufacturer’s instructions (Maixin-Bio Corporation, Fuzhou, China). The dilution ratio of anti-VEGF, anti-VEGF-2 and anti-p-Akt1(Abcam) was 1:200. The dilution ratio of anti-Akt1, anti-mTOR(Abcam), and anti-p-mTOR (Cell Signaling Technology) was 1:100.

### Western blotting analysis

The total protein from ectopic endometria in each group was extracted, and 80 μg of total protein from each group was loaded per lane on a 10% polyacrylamide geland subjected to electrophoresis. The proteins were transferred to polyvinylidene difluoride membranes. After transfer, the membranes were blocked for 30 min in a 5% BSA buffer (Shanghai Bocai Corporation or NEB). The membranes were then incubated with VEGF and VEGFR-2 polyclonal antibodies, and with AKT1, anti-p-Akt1, mTOR andanti-p-mTOR monoclonal antibodiesin blocking buffer (diluted 1:800 with 5% BSA) for 90 min at 37°C. After three washes with PBST, the membranes were incubated with HRP-conjugated goat anti-rabbit and goat anti-rat secondary antibodies (diluted1:2000 with 5% BSA) for 60 min at 37°C. The primary β-actin antibody was diluted 1:5000 with 5% BSA and was incubated with HRP-conjugated goat anti-rabbit and goat anti-rat secondary antibodies for 1 h. The membranes were washed three times with PBST, and photographs were taken immediately after color development using a Gel Imaging System (Bio-Rad Laboratories, Inc., Hercules, California, USA). The relative levels of protein were semi-quantitatively determined using Quantity One band analysis software.

### RNA isolation and real-timePCR

Total RNA was isolated from ectopic endometrial tissue using TRIzol (Invitrogen, Carlsbad, CA, USA),and cDNA synthesis was performed using the reverse transcriptase amplification kit (Fermentas, New York,USA).mRNA levels were evaluated by real-time PCR using SYBR Premix Ex Taq (Takara, Takara Bio, Inc., Otsu, Shiga,Japan). Expression values were normalized against the expression of the reference gene GAPDH, and the quantification of mRNA abundance was made using the method described in Livak and Schmittgen(2001). The gene targets and their primers are listed in S2 Table.

### Terminal DeoxynucleotidylTransferase-mediated Digoxigenin-dUTPNick-End Labeling Assay (TUNEL)

The paraffin-embedded tissue sections were dewaxed in xylene and rehydrated after remaining in a 60°C warm case for 1 h. The sections were washed three times, for 5 minutes per wash, with TBS. The following operations were conducted according to manufacturer’s instructions (Merck Corporation, U.S.A.). Next, a 2 mg/ml proteinase K stock solution was diluted with 10mMTris solution (pH 8.0) to a final concentration of 20 μg/ml. A volume of 100 μl of diluted proteinase K was added to each sample and incubated for 20 min at 37°C. The sections were washed three times with TBS for 5 min each wash. Distilled water was used to dilute the 5×TdT balance buffer. To each sample, 100 μl 1×TdT was added as a balance buffer and incubated for 20 min at 37°C. A volume of 3 μl of terminal deoxynucleotidyl transferase (TdT) was stored in the refrigerator and then removed and mixed with 57 μl fluorescein labeling reaction mixture. A balance buffer of 1×TdT around the samples was blotted up, and 60 μl TdT labeling reaction mixture was added to each sample and incubated for 60 minutes at 37°C. The sections were washed three times with TBS. Samples were mounted on slides and treated with mounting media. The labeled apoptotic cells expressed green fluorescence under fluorescence microscopy.

### Statistical analysis

The statistical analysis was conducted in SPSS 18.0 software (version 18.0 for Windows; SPSS, Chicago, Illinois, USA). Data were initially tested for normal distribution. Data with a normal distribution were analyzed by one-way ANOVA using the LSD or Games-Howell methods and was presented as mean ± SD Data that did not conform to a normal distribution were presented as Median range[M±Q]. The statistical significance among three or more groups was determined using Kruskal-Wallis H analysis. Mann-Whitney U test was performed to compare between two groups. *P* < 0.05 was considered to be statistically significant.

## Results

### Effect of ginsenoside Rg3 on weight change in rats

**Fig 2:**
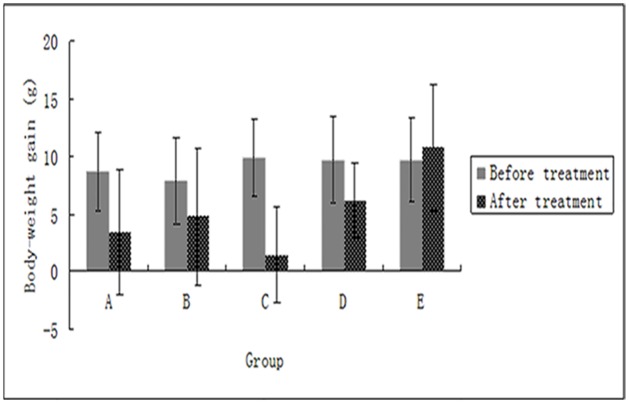
Weekly weight change in rats in before and after the treatment (n = 12). Weight is represented as mean ± SD. (A: Ginsenoside Rg3 low-dosage group; B: Ginsenoside Rg3 high-dosage group; C: Gestrinone group; D: Model control group; E: Ovariectomized group).

Rats in each group were weighed weekly during the treatment, and the increases in weight were calculated. After the treatment, the weekly weight of the ovariectomized group clearly increased, whereas the gestrinone group showed negative growth. The ginsenosideRg3 groups and the model control group exhibited varying decreases in weekly weight. There was no significant difference in the increase of weight in post-treatment between groups. This suggests that the two dosages of ginsenoside Rg3 administered had no toxic side effects on rats(S3 Table,Fig 2).

### Effect of ginsenosideRg3 on growth inhibition rate in rats

**Fig 3:**
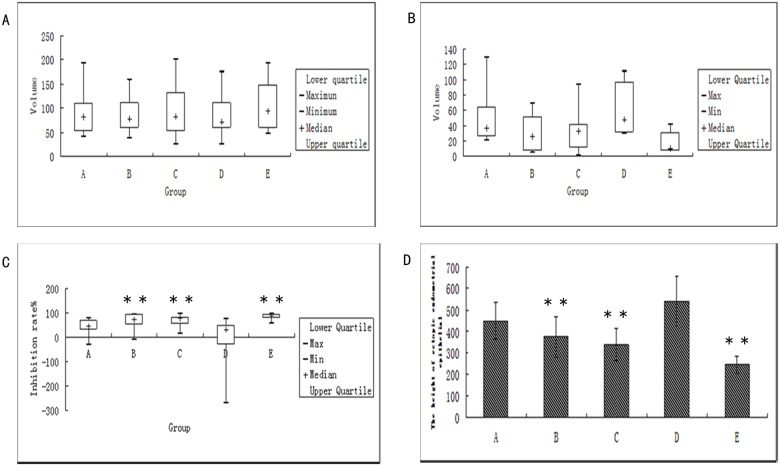
Inhibitory effect of ginsenosideRg3 on ectopic endometrial growth. (a) The volume of ectopic endometrial before treatment (n = 12); (b) The volume of ectopic endometrial after treatment (n = 12); (c) The inhibition rate of ectopic endometrialafter treatment (n = 12); **: P<0.01, *:P<0.05 (by Mann-Whitney U test). Volume is presented in median range (d) The epithelial height of ectopic endometrialafter treatment (n = 6). **: P<0.01 (by LSDt-test). Height is presented as means ± SDs. (A: Ginsenoside Rg3 low-dosage group; B: Ginsenoside Rg3 high-dosage group; C: Gestrinone group; D: Model control group; E: Ovariectomized group).

The volumes of ectopic endometrial lesions in each group pre- (V1) and post- (V2) treatment were measured, and the inhibitory rate was calculated according to the following formula: inhibitory rate (%) = (V1-V2) / V1×100%. The volumes of ectopic endometrial lesions in each group were reduced to different degrees after treatment, and pronounced reductions were observed in the ovariectomized group, gestrinone group, and ginsenoside Rg3 high-dosage group compared with the model control group (P<0.05). However, there was no significant difference between the gestrinone group and the ginsenoside Rg3 high-dosage group (S4 Table,Fig 3).

### Effect of ginsenoside Rg3 on the ectopic endometrial epithelial height

The greatest height in the ectopic endometria was observed in the model control group. The heights of the ectopic endometria of the ginsenoside Rg3 high-dose group, gestrinone group, and ovariectomized group were significantly lower than those of the model control group(P<0.01)(S5 Table).

### Effect of ginsenoside Rg3 on the levels of serum estrogen (E2) and progesterone (P)

**Fig 4:**
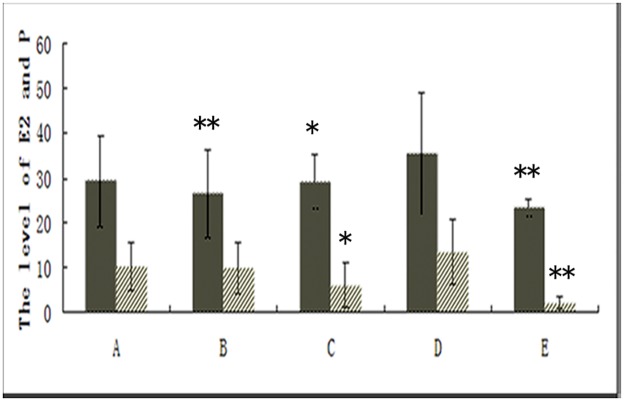
Effect of ginsenoside Rg3 on the levels of E_2_ (left panel) and P (right panel) in serum as assessed by the ECLI assay (n = 12). **: P<0.01, *:P<0.05 (by LSDt-test). Data are represented in means ± SDs. (A: Ginsenoside Rg3 low-dosage group; B: GinsenosideRg3 high-dosage group; C: Gestrinone group; D: Model control group; E: Ovariectomized group).

After treatment for three weeks, the levels of serum estrogen (E_2_) and progesterone (P) in each group had decreased to different degrees (S6 Table, Fig 4). The levels of serumE_2_ in the ginsenoside Rg3 high-dosage group, gestrinone group,and ovariectomized group clearly decreased compared with the model control group (P<0.05). Compared with the model control group, the levels of serum P in the gestrinone group and in the ovariectomized group showed a significant decrease(P<0.05). However, there was no significant difference between both the ginsenoside Rg3 groups and the model control group.

### Effect of ginsenoside Rg3 on the levels of protein expression and localization of VEGF, VEGFR-2, p-Akt and p-mTOR in the ectopic endometria as measured by immunohistochemically

**Fig 5:**
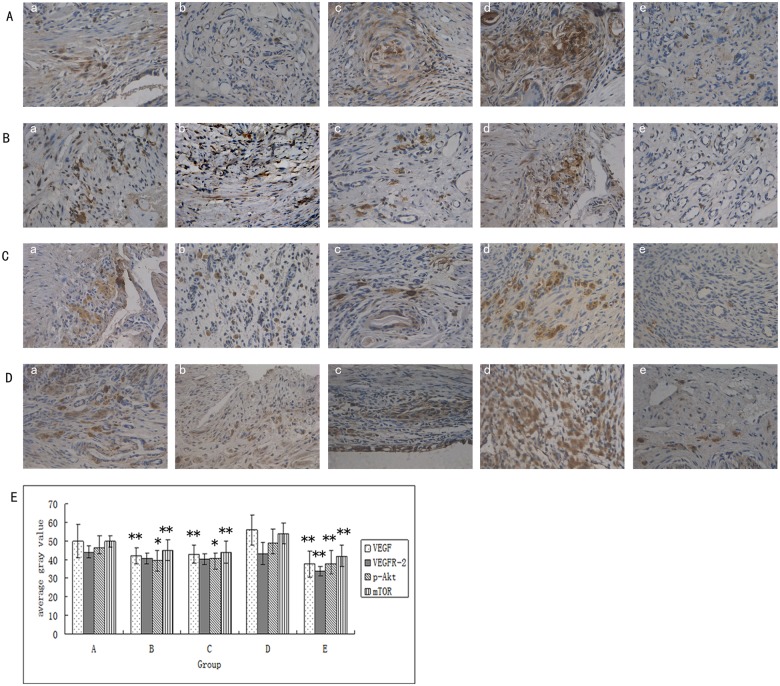
Effect of ginsenoside Rg3 on the protein expression levels and localization of VEGF, VEGFR-2, p-Akt, p-mTOR in the ectopic endometriaas measured by immunohistochemically. (a) Protein expression and localization of VEGF, VEGFR-2, p-Akt, p-mTOR in the ectopic endometria by DAB staining, as assessed via electron microscopy. All magnifications were ×400. (b) Effect of ginsenoside Rg3 on VEGF, VEGFR-2, p-Akt, p-mTOR protein expression levels in the ectopic endometria (n = 6) as measured by the average gray values,**: P<0.01, *:P<0.05 (by LSD t-test). Average gray values are presented as mean ± SD. (A: Ginsenoside Rg3 low-dosage group; B: Ginsenoside Rg3 high-dosage group; C: Gestrinone group; D: Model control group; E: Ovariectomized group).

The immunohistochemical results showed VEGF and VEGFR-2 expression located in the glandular epithelial cells, stromal cellular cytoplasm and vascular endothelial cells of the ectopic endometria, with strong expression in the glandular epithelial cells. p-Akt and p-mTOR proteins were mainly expressed in the cytoplasm of endometrial stromal cells, with some expression in the cytoplasm of the glandular epithelium. The average gray values of VEGF, p-Akt, and p-mTOR in the ginsenoside Rg3 high-dose group, gestrinone group, and ovariectomized group were significantly reduced compared to those of the model control group (P<0.05). Apart from the ovariectomized group, there was no obvious difference in the average gray values of VEGFR-2 in the other treatment groups as compared with the model control group (P>0.05) (S7 Table,Fig 5).

### Effect of ginsenosideRg3 on the VEGF,VEGFR-2,p-Akt, and p-mTOR protein expression levels in the ectopic endometria as assessed by Western blotting

**Fig 6:**
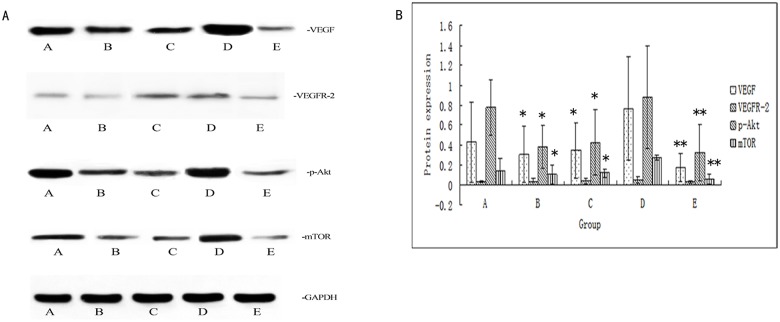
Effect of ginsenoside Rg3 on the protein expression levels of VEGF, VEGFR-2, p-Akt, p-mTOR in the ectopic endometria as assessed by Western blotting. (a) Representative Western blotting results for VEGF, VEGFR-2, p-Akt, p-mTOR protein expression. (b) Relative fold change in protein expression levels of VEGF, VEGFR-2, p-Akt, p-mTOR in the ectopic endometria (n = 6). **: P<0.01, *:P<0.05 (by LSD t-test). Data are represented in mean ± SD. (A: GinsenosideRg3 low-dosage group; B: Ginsenoside Rg3 high-dosage group; C: Gestrinone group; D: Model control group; E: Ovariectomized group).

Western blotting results showed that the expression of VEGF, p-Akt, and p-mTOR in the ectopic endometrial in each group was down-regulated after treatment. There was a significant difference in the protein expression level of VEGF between the model control group and the ginsenosideRg3 high-dosage group(P<0.05), which is consistent with the immunohistochemical results. However, ginsenoside Rg3 had no effect on the expression of total Akt and mTOR protein(S8 Table,Fig 6).

### Effect of ginsenoside Rg3 on gene expression levels of VEGF,VEGFR-2, Akt, and mTOR

**Fig 7:**
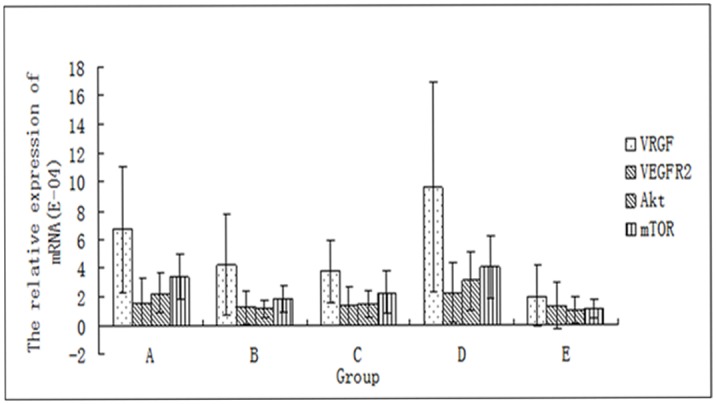
Effect of ginsenoside Rg3 on gene expression levels of VEGF, VEGFR-2, Akt, mTOR against reference gene in the ectopic endometria (n = 6). (A: Ginsenoside Rg3 low-dosage group; B: Ginsenoside Rg3 high-dosage group; C: Gestrinone group; D: Model control group; E: Ovariectomized group).

The extracted VEGF, VEGFR-2, Akt and mTOR genes were amplified by real-time PCR. The relative expression in ectopic endometrial cells from each group is shown in S9 Table. In addition to VEGFR-2, the gene expression levels of VEGF, Akt and mTOR in the ectopic endometrial in the ginsenoside Rg3 high-dosage, gestrinone, and ovariectomized groups were significantly reduced compared to the model control group(P<0.05) (S9 Table,Fig 7).

### Effect of ginsenoside Rg3 on the apoptotic morphological features of ectopic endometria

**Fig 8:**
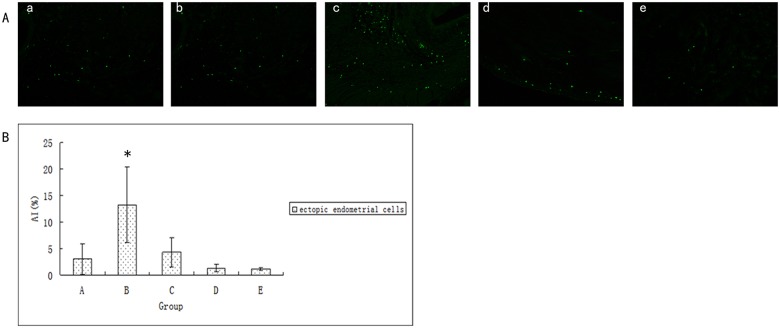
Effect of ginsenoside Rg3 on the apoptotic morphological features of ectopic endometria. (a) Apoptosis in ectopic endometria was observed by TUNEL assay. Green color represents TUNEL-positive cells as apoptosis. (b) The apoptotic index of ectopic endometrial cells after treatment (n = 6). *:P<0.05 (by LSD t-test). Data are represented in means ± SDs. (A: Ginsenoside Rg3 low-dosage group; B: Ginsenoside Rg3 high-dosage group; C: Gestrinone group; D: Model control group; E: Ovariectomized group).

Apoptosis occurred in the ectopic endometrial stroma of each group after 21 days of drug administration. Compared with the model control group, granular loosening of nucleosomes and nuclear cracking phenomena were obvious in the ginsenoside Rg3 high-dosage group. This result indicates that ginsenoside Rg3 can promote the apoptosis of ectopic endometrial tissues. Simultaneously, we observed that most ectopic endometrial stromain of the ovariectomized group had atrophied and did not exhibit obvious immunofluorescent labeling (S10 Table,Fig 8). This suggests that the intensity of apoptosis was related to the rich degree of ectopic endometrial stroma richness.

## Discussion

In an effort to clarify the pathological angiogenesis in endometriosis and to establish novel therapeutic strategies for this disease, our laboratory has been conducting an ongoing investigation into the mechanism of traditional Chinese medicine (TCM) drug that acts against angiogenesis in endometriosis. In the current study, we demonstrated that ginsenoside Rg3 can significantly inhibit protein and gene expression of VEGF in the ectopic endometrium and reduce phosphorylation of Akt and mTOR, proteins thereby inhibiting the PI3K/Akt/mTOR signal transduction cascade, and halting angiogenesis. This finding suggests that the PI3K-Akt-mTOR signaling pathway is involved in the pathogenesis of endometriosis-associated angiogenesis and that ginsenoside Rg3 might be a drug target for the treatment and prevention of endometriosis-associated angiogenesis.

At present, the etiology and pathophysiology of EMs are unclear. For endometrium to develop into an endometriosis lesion, endometrial cells must be stimulated to proliferate, adhere and invade into the peritoneum. Once established, the lesion must develop a vascular network to support itsnutrient and oxygen demands. There is evidence that the development of this vascular network occurs by angiogenesis, with the formation of blood vessels from preexisting blood vessels being important for EMs progression[7].

As an important intracellular signal transduction pathway mediated by VEGFR2, the phosphatidylinositol-3 kinase (PI3K)-Akt-mammalian target of rapamycin (mTOR) pathway is a key cellular signaling pathway that affects multiple cellular functions, including metabolism, growth, proliferation, and apoptosis. Because of the change in the peritoneal microenvironment in EMs patients, the ability of ectopic endometrial cells to tolerate hypoxia and lack of nutrition is important for their proliferation and metastasis. Akt plays a key role in helping cells adapt to these adverse environments and maintain proliferation. When Akt is activated by VEGF, it can prevent the apoptosis of endothelial cells. Phosphorylated Akt was higher in the ectopic endometrium than in the normal endometrium, and estrogen may be one of the factors that influence the activation of Akt in ectopic endometrial cells[15]. Leconte et al.[16]observed that the proliferation rate of eutopic and ectopic endometrial cells in deep infiltrating EMs patients was significantly higher. The over-proliferative phenotype of ectopic endometrial cells and the increase inendogenous oxidative stress are associated with activation of the mTOR/Akt pathway, and conversely, a mTOR/Akt inhibitor can effectively inhibit the proliferation of ectopic endometrial cells. Rapamycin is an inhibitor of mTOR, and the study showed that it could induce ectopic endometrium apoptosis by inhibiting angiogenesis and cell proliferation[17]. The researchers also found that tumor metastasis inhibitory factor NME1 can regulate the angiogenesis of EMs. The abnormally low expression of NME1 in endometrial interstitial cells can activate the PI3K/Akt pathway, resulting in increased protein expression of IL-8 and VEGF as well as of the angiogenesis-related molecules CD62E and CD105 in order to promote mass production of vascular endothelial cells in ectopic endometrial lesions[18]. Akt promotes the phosphorylation of endothelial nitric oxide synthase (eNOS) generating NO, which could stimulate angiogenesis[19,20]. The expression of eNOS and the angiogenesis of the ectopic endometrium in patients with EMs was significantly increased, which may be related to the activation of the PI3K/Akt pathway[21]. In addition, the Akt pathway can also be combined with the ERK pathway to promote the growth of deep endometriosis through enhancing the proliferation of ectopic endometrial stromal cells and their viability in the fibrotic microenvironment[22]. In conclusion, the phosphorylation of a variety of proteins in the PI3K-Akt-mTOR pathway leads to molecule interactions, which ultimately lead to the formation of EMs.

Consequently, the application of angiogenesis inhibitors has unique advantages in the treatment of EMs, as endothelial cells are more readily accessible to blood diffused drugs than other cells. The vessels in ectopic endometria lesions in EMs are different from normal vessels, as most of the vessels that supply blood to the ectopic endometrium are immature and lack surrounding cells. The microvessel density in the peritoneal red lesions is higher than in brown and white lesions; most of these vessels are immature lacking smooth muscle protection[23]. The administration of angiogenesis inhibitors can inhibit the growth of immature vessels in the early stage, but the effect on mature vessels is weak[24]. Ferrara et al[25] found that the physiologically mature vessels were not affected by vascular inhibitors in EMs rat models, and that angiogenesis inhibitors have minimal toxicity to normal tissues[26].

Therefore, the present study relies on the assumptions that angiogenesis inhibitors can be used in the early treatment of endometriosis and have an ability to prevent recurrence. It also foresees their possible use in eliminating residual lesions after laparoscopic surgery. Furthermore, it assumes a role for angiogenesis inhibitors in the treatment of EMs-associated infertility, as its administration does not interfere with ovulation or has any other fertility-related side effects.

As a type of anti-tumor inhibitor, ginsenoside Rg3 is known to inhibit tumor cell proliferation, reduce adhesion and tumor invasion, and slow-down tumor angiogenesis, the latter being the specific effect on EMs[11]. Our study found that ginsenoside Rg3 can reduce the level of serum E_2_ in rats and inhibit the growth of the ectopic endometrium. The Western blotting results showed that the high-dose ginsenoside Rg3 group exhibited reduced protein expression of VEGF, p-Akt, and p-mTOR in the ectopic endometrium but had no effect on the expression of total Akt and mTOR protein levels. Real-time quantitative PCR assays also showed that mRNA expression of VEGF, Akt and mTOR were down-regulated in the ectopic endometrium of rats after treatment, with the administration of high-dose ginsenoside Rg3 and of gestrinone having a similar effect. The TUNEL results showed that compared with the model control group, the number of fluorescent cells in the ectopic endometrium of the high-dose ginsenoside Rg3 group was significantly increased, which is negatively correlated with the number of apoptotic cells. This suggests that the VEGF binding to VEGFR-2 in vascular endothelial cells after endometrium anarrhea into the abdominal cavity might have activated the downstream PI3K/Akt/mTOR signaling pathway, therefore, increasing vascular permeability, enhancing the invasion ability of the ectopic endometrial cells and angiogenesis, promoting cell proliferation and reducing cell apoptosis to eventually cause EMs. In addition, EMs patients have a high estrogen status. Studies found that 17β-E_2_ types could activate the PI3K/Akt pathway to promote EMs cell proliferation by regulating the activity of NF-kB/PTEN. Same studies also suggested that there may be a positive feedback loop in this process where 17β-E2 activated NF-kB which then inactivated PTEN. The inactivation of PTEN was conducive to the activation of PI3K, and excessive activation of PI3K continued to promote the activation of NF-kB, thereby promoting the production of VEGF[27]. Ginsenoside Rg3 can reduce the expression of VEGF, decrease the level of serum estrogen, and reduce the phosphorylation of Akt and mTOR, thereby inhibiting the PI3K/Akt/mTOR signal transduction cascade mediated by VEGFR-2. This resulted in the restraining of angiogenesis caused by the cutting-off of the source of nutrition and the blocking of the migration pathway of the ectopic endometrium necessary for growth and metastasis. The overall consequences of this angiogenesis inhibitory mechanism are reduced growth and planting ability, and the promotion the apoptosis of the ectopic endometrial cells. This may be the mechanism underlying the effect of ginsenoside Rg3 on the treatment of EMs. However, other molecular pathways involved in the pathogenesis of EMs remain explored.

## Supporting information

S1 Table Experimental design. (DOC)

S2 Table List of primers used in the real-time PCR analysis. (DOCX)

S3 Table Weekly weight change in rats before and after treatment. (DOCX)

S4 Table Effect of ginsenoside Rg3 on ectopic endometrial growth in rats. (DOCX)

S5 Table Effect of ginsenoside Rg3 on the ectopic endometrial epithelial height. (DOCX)

S6 Table Post-treatment levels of serum E2 and P as assessed by the ECLI assay. (DOCX)

S7 Table Effect of ginsenosideRg3 on the protein expression levels of VEGF,VEGFR-2, p-Akt and p-mTOR in the ectopic endometriaas measured by immunohistochemically. (DOCX)

S8 Table Effect of ginsenoside Rg3 on the protein expression levels of VEGF,VEGFR-2, p-Akt, and p-mTORas assessed by Western blotting. (DOCX)

S9 Table Effect of ginsenoside Rg3 on relative gene expression levels of VEGF,VEGFR-2, Akt,and mTOR against reference gene. (DOCX)

S10 Table Effect of ginsenoside Rg3 on the apoptotic morphological features of ectopic endometria. (DOCX)

S11 Table Supporting data-weekly weight change. (XLSX)

S12 Table Supporting data-ectopic endometrial volume. (XLSX)

S13 Table Supporting data-ectopic endometrial epithelial height. (XLS)

S14 Table Supporting data-serum E2 and P. (XLS)

S15 Table Supporting data-IHC. (XLSX)

S16 Table Supporting data-WB. (XLS)

S17 Table Supporting data-PCR. (XLS)

S18 Table Supporting data-tunel. (XLS)
