# Supplementary material for: Ginsenoside Rg3 inhibits angiogenesis in a rat model of endometriosis through the VEGFR-2-mediated PI3K/Akt/mTOR signaling pathway
Source: PLoS One. 2017 Nov 15;12(11):e0186520. doi: 10.1371/journal.pone.0186520 (PMC5687597; doi:10.1371/journal.pone.0186520)
Supplement: S1 Table — (DOC) [file pone.0186520.s001.doc]

**Table 1. Experimental Design**

| **Group** | **Drug** | **N** | **Dosage** |
| --- | --- | --- | --- |
| ginsenoside Rg3 low-dosage group | ginsenoside | 12 | 5mg/(kg·d)×21d |
| ginsenoside Rg3 high-dosage group | Rg3 ginsenoside Rg3 | 12 | 10 mg/(kg·d)×21d |
| gestrinone group | gestrinone Rg3 | 12 | 0.5mg/(kg·d)×21d |
| model control group | 0.5%CMC | 12 | 5 ml/(kg·d)×21d |
| ovariectomized group | 0.5%CMC | 12 | 5 ml/(kg·d)×21d |
