# Supplementary material for: Ginsenoside Rg3 inhibits angiogenesis in a rat model of endometriosis through the VEGFR-2-mediated PI3K/Akt/mTOR signaling pathway
Source: PLoS One. 2017 Nov 15;12(11):e0186520. doi: 10.1371/journal.pone.0186520 (PMC5687597; doi:10.1371/journal.pone.0186520)
Supplement: S2 Table — (DOCX) [file pone.0186520.s002.docx]

**Table2.List of Primers used in the Real-Time PCR Analysis**

| **Gene name** | **Sequence** |  |  |  |
| --- | --- | --- | --- | --- |
| VEGF | forward | 5’-TTCAGAGCGGAGAAAGCAT-3’ | | |
|  | reverse | 5’-TAGTTCCCGAAACCCTGAG-3’ | | |
| VEGFR-2 | forward | 5’-GAATGCGGGCTCCTGACTAC-3’ | | |
|  | reverse | 5’-GAAACAGGTGAGGTAGGCAGG-3’ | | |
| Akt | forward | 5’-CGCCTGCCCTTCTACAACC-3’ | | |
|  | reverse | 5’-TCATACACATCTTGCCACACGA-3’ | | |
| mTOR | forward | 5’-TTGGAGAACCAGCCCATAAGA-3’ | | |
|  | reverse | 5’-ATGAGATGTCGCTTGCTTGATAA-3’ | | |
| GAPDH | forward | 5’-AGTGCCAGCCTCGTCTCATAG-3’ | |  |
|  | reverse | 5’-CGTTGAACTTGCCGTGGGTAG-3’ | |  |
