# Supplementary material for: Ginsenoside Rg3 inhibits angiogenesis in a rat model of endometriosis through the VEGFR-2-mediated PI3K/Akt/mTOR signaling pathway
Source: PLoS One. 2017 Nov 15;12(11):e0186520. doi: 10.1371/journal.pone.0186520 (PMC5687597; doi:10.1371/journal.pone.0186520)
Supplement: S3 Table — (DOCX) [file pone.0186520.s003.docx]

**Table3.Weekly Weight Change in Rats Before and After Treatment ($\overline{\text{X}}\pm\text{S}$)**

| Groups | N | Pre-treatment (g) | Post-treatment (g) |
| --- | --- | --- | --- |
| ginsenoside Rg3 low-dosage group (A) | 12 | 8.78±3.40 | 3.44±5.37 |
| ginsenoside Rg3 high-dosage group (B) | 12 | 7.86±3.76 | 4.81±5.95 |
| gestrinone group(C) | 12 | 9.86±3.36 | 1.50±4.17* |
| model control group (D) | 12 | 9.67±3.78 | 6.19±3.24 |
| ovariectomized group (E) | 12 | 9.00±3.64 | 10.81±5.46* |

*P<0.05,compared with the model control group
