# Supplementary material for: Ginsenoside Rg3 inhibits angiogenesis in a rat model of endometriosis through the VEGFR-2-mediated PI3K/Akt/mTOR signaling pathway
Source: PLoS One. 2017 Nov 15;12(11):e0186520. doi: 10.1371/journal.pone.0186520 (PMC5687597; doi:10.1371/journal.pone.0186520)
Supplement: S4 Table — (DOCX) [file pone.0186520.s004.docx]

**Table4.Effect of ginsenoside Rg3 on ectopic endometrial growth in rats**

| Groups | N | Ectopic endometrial volume (mm^3^) | | Inhibition rate % |
| --- | --- | --- | --- | --- |
|  |  | Pre-treatment | Post-treatment |  |
| ginsenoside Rg3 low-dosage group (A) | 12 | 83.47±56.65 | 37.20±38.18 | 45.05±35.97 |
| ginsenoside Rg3 high-dosage group (B) | 12 | 77.38±53.14 | 26.01±44.81^*^ | 70.29±40.58^**^ |
| gestrinone group(C) | 12 | 83.52±80.15 | 32.57±31.34^*^ | 75.95±27.68^**^ |
| model control group (D) | 12 | 71.39±52.64 | 48.41±65.76 | 31.15±76.68 |
| ovariectomized group (E) | 12 | 93.70±88.38 | 10.38±24.22^**^ | 85.25±13.49^**^ |

^**^P＜0.01，^*^P＜0.05（compared with the model control group）
