# Supplementary material for: Ginsenoside Rg3 inhibits angiogenesis in a rat model of endometriosis through the VEGFR-2-mediated PI3K/Akt/mTOR signaling pathway
Source: PLoS One. 2017 Nov 15;12(11):e0186520. doi: 10.1371/journal.pone.0186520 (PMC5687597; doi:10.1371/journal.pone.0186520)
Supplement: S5 Table — (DOCX) [file pone.0186520.s005.docx]

**Table.5 Effect of ginsenosideRg3 on the ectopic endometrial epithelial height**

| Group | N | Height of ectopic endometrial epithelial（μm） |
| --- | --- | --- |
| ginsenoside Rg3 low-dosage group (A) | 6 | 447.87 ±86.19 |
| ginsenoside Rg3 high-dosage group (B) | 6 | 375.78±93.79^**^ |
| gestrinone group(C) | 6 | 337.62±74.19^**^ |
| model control group (D) | 6 | 539.06±115.06 |
| ovariectomized group (E) | 6 | 245.87±38.77^**^ |

^**^P＜0.01，compared with the model control group
