# Supplementary material for: Ginsenoside Rg3 inhibits angiogenesis in a rat model of endometriosis through the VEGFR-2-mediated PI3K/Akt/mTOR signaling pathway
Source: PLoS One. 2017 Nov 15;12(11):e0186520. doi: 10.1371/journal.pone.0186520 (PMC5687597; doi:10.1371/journal.pone.0186520)
Supplement: S6 Table — (DOCX) [file pone.0186520.s006.docx]

**Table6. Post-Treatment levels of serum E_2_ and P as assessed by the ECLI assay**

| Group | N | | E_2_(pg/ml) | P(ng/ml) |
| --- | --- | --- | --- | --- |
| ginsenoside Rg3 low-dosage group (A) | 12 | | 29.31±7.66 | 10.27±5.41 |
| ginsenoside Rg3 high-dosage group (B) | 12 | | 26.46±6.44^**^ | 9.89±5.66 |
| gestrinone group(C) | 12 | | 28.96±10.75^*^ | 6.05±4.82^*^ |
| model control group (D) | 12 | | 35.55±8.08 | 13.47±6.98 |
| ovariectomized group (E) | | 12 | 23.26±4.45^**^ | 2.02±1.36^**^ |

^**^P＜0.01，^*^P＜0.05（compared with the model control group）
