# Supplementary material for: Ginsenoside Rg3 inhibits angiogenesis in a rat model of endometriosis through the VEGFR-2-mediated PI3K/Akt/mTOR signaling pathway
Source: PLoS One. 2017 Nov 15;12(11):e0186520. doi: 10.1371/journal.pone.0186520 (PMC5687597; doi:10.1371/journal.pone.0186520)
Supplement: S7 Table — (DOCX) [file pone.0186520.s007.docx]

**Table 7Effect of ginsenosideRg3 on the protein expression levels of VEGF,VEGFR-2, p-Akt and p-mTOR in the ectopic endometrial as measured by immunohistochemically**

| Group | N | VEGF | VEGFR-2 | p-Akt | p-mTOR |
| --- | --- | --- | --- | --- | --- |
| ginsenoside Rg3 low-dosage group (A) | 6 | 49.87±8.97 | 44.11±3.22 | 46.40±6.37 | 49.72±2.98 |
| ginsenoside Rg3 high-dosage group (B) | 6 | 42.25±4.41^**^ | 40.82±2.94 | 39.42±5.72^*^ | 45.19±5.52^**^ |
| gestrinone group(C) | 6 | 42.88±4.62^**^ | 40.33±3.01 | 40.70±3.04^*^ | 44.00±5.99^**^ |
| model control group (D) | 6 | 56.02±8.20 | 43.28±5.90 | 48.83±7.61 | 53.97±5.69 |
| ovariectomized group (E) | 6 | 37.65±7.11^**^ | 33.64±2.55^**^ | 37.79±7.15^**^ | 41.86±5.66^**^ |

^**^P＜0.01，*P＜0.05（compared with the model control group）
