# Supplementary material for: Ginsenoside Rg3 inhibits angiogenesis in a rat model of endometriosis through the VEGFR-2-mediated PI3K/Akt/mTOR signaling pathway
Source: PLoS One. 2017 Nov 15;12(11):e0186520. doi: 10.1371/journal.pone.0186520 (PMC5687597; doi:10.1371/journal.pone.0186520)
Supplement: S8 Table — (DOCX) [file pone.0186520.s008.docx]

**Table8.Effect of ginsenoside Rg3 on the protein expression levels of VEGF,VEGFR-2, p-Akt, and p-mTOR as assessed by Western blotting**

| Group | N | VEGF | VEGFR-2 | p-Akt | p-mTOR |
| --- | --- | --- | --- | --- | --- |
| ginsenoside Rg3 low-dosage group (A) | 6 | 4.30±4.05 E-01 | 3.75±1.04E-02 | 7.77±2.79 E-01 | 1.35±1.3 E-01 |
| ginsenoside Rg3 high-dosage group (B) | 6 | 3.04±2.79 E-01^*^ | 3.52±3.54E-02 | 3.79±2.17 E-01^*^ | 1.05 ±0.91 E-01^*^ |
| gestrinone group(C) | 6 | 3.46±2.76 E-01^*^ | 4.09±3.32E-02 | 4.25±3.30 E-01^*^ | 1.18±0.39E-01^*^ |
| model control group (D) | 6 | 7.67±5.20 E-01 | 5.5±3.6E-02 | 8.79±5.17 E-01 | 2.73±2.24E-01 |
| ovariectomized group (E) | 6 | 1.76±1.41 E-01^* *^ | 3.26±1.53E-02 | 3.21±2.80 E-01^**^ | 5.9 ±4.67E-02^**^ |

^* *^P<0.01,^*^P＜0.05（compared with the model control group）
