# Supplementary material for: Ginsenoside Rg3 inhibits angiogenesis in a rat model of endometriosis through the VEGFR-2-mediated PI3K/Akt/mTOR signaling pathway
Source: PLoS One. 2017 Nov 15;12(11):e0186520. doi: 10.1371/journal.pone.0186520 (PMC5687597; doi:10.1371/journal.pone.0186520)
Supplement: S9 Table — (DOCX) [file pone.0186520.s009.docx]

**Table9. Effect of ginsenoside Rg3 on relative gene expression levels of VEGF,VEGFR-2, Akt,and mTOR against reference gene**

| Group | N | VEGF | VEGFR-2 | Akt | mTOR |
| --- | --- | --- | --- | --- | --- |
| ginsenoside Rg3 low-dosage group (A) | 6 | 6.69±4.39E-04 | 1.59±1.66E-04 | 2.25±1.35 E-04 | 3.39±1.56E-04 |
| ginsenoside Rg3 high-dosage group (B) | 6 | 4.22±3.5 E-04^*^ | 1.24±1.2E-04 | 1.16±0.61 E-04^*^ | 1.84±0.94 E-04^*^ |
| gestrinone group(C) | 6 | 3.73±2.18 E-04^*^ | 1.36±1.38E-04 | 1.50±0.92E-04^*^ | 2.27±1.49E-04^*^ |
| model control group (D) | 6 | 9.57±7.29 E-04 | 2.24±2.04E-04 | 3.03±2.05 E-04 | 4.05±2.16E-04 |
| ovariectomized group (E) | 6 | 1.99±2.08E-04^* *^ | 1.32±1.63E-04 | 1.03±0.93E-01^*^ | 1.1±0.68E-04^**^ |

^* *^P<0.01,^*^P＜0.05（compared with the model control group）
