# Supplementary material for: Ginsenoside Rg3 inhibits angiogenesis in a rat model of endometriosis through the VEGFR-2-mediated PI3K/Akt/mTOR signaling pathway
Source: PLoS One. 2017 Nov 15;12(11):e0186520. doi: 10.1371/journal.pone.0186520 (PMC5687597; doi:10.1371/journal.pone.0186520)
Supplement: S10 Table — (DOCX) [file pone.0186520.s010.docx]

**Table10.Effect of ginsenoside Rg3 on the apoptotic morphological features of ectopic endometria**

| Group | N | Apoptotic index(%) |
| --- | --- | --- |
| ginsenoside Rg3 low-dosage group (A) | 6 | 3.02±2.83 |
| ginsenoside Rg3 high-dosage group (B) | 6 | 13.25±7.11* |
| gestrinone group(C) | 6 | 4.27±2.74 |
| model control group (D) | 6 | 1.35±0.64 |
| ovariectomized group (E) | 6 | 1.16±0.29 |

^*^P＜0.05（compared with the model control group）
